# Rebound or Retention: A Meta-Analysis of Weight Regain After the Discontinuation of Glucagon-Like Peptide-1 (GLP-1) Receptor Agonists and Other Anti-obesity Drugs

**DOI:** 10.7759/cureus.94926

**Published:** 2025-10-19

**Authors:** Ravi Teja Kolli, Sridevi Aoutla, Nirmal Jyothi, Mohamed Raghib Hussain Mohamed Kalifa, Arvin Raju, Kavya Cheenikkal Muralidharan

**Affiliations:** 1 Department of General Medicine, Chettinad Institute of Medical Sciences, Chennai, IND; 2 Department of Obstetrics and Gynecology, University Hospitals Sussex NHS Foundation Trust, Worthing, GBR; 3 Department of Critical Care Medicine, Max Hospital, Dehradun, IND; 4 Department of Emergency Medicine, University Hospital Southampton NHS Foundation Trust, Southampton, GBR; 5 Department of General Medicine, Arthurs Hospital, Nagapattinam, IND; 6 Department of Cardiology, Manchester University NHS Foundation Trust, Manchester, GBR

**Keywords:** anti-obesity medications, glp-1 receptor agonists, meta-analysis, orlistat, weight retention

## Abstract

Anti-obesity pharmacotherapies, such as glucagon-like peptide-1 (GLP-1) receptor agonists and orlistat, are effective for weight loss; however, weight regain following treatment discontinuation remains a major concern. This meta-analysis aimed to quantify the magnitude of weight regain after reaching peak weight loss, and to compare rebound effects across four commonly used agents: semaglutide, liraglutide, exenatide, and orlistat.

A systematic search was conducted in PubMed (n = 498), Cochrane Library (n = 41), Scopus (n = 258), ScienceDirect (n = 248), and Web of Science (n = 158) from January 2010 to October 2024. After removing 153 duplicates, 950 records were screened. Following full-text assessment, 36 studies were included in the final analysis. Data extraction was performed using Excel (Microsoft® Corp., Redmond, WA, USA), and graphical data were digitized using WebPlotDigitizer. Risk of bias was assessed using RoB 2.0, and meta-analyses were conducted with Comprehensive Meta-Analysis (v3.7), using mean difference (MD) and 95% confidence intervals (CI). I² statistics were used to assess heterogeneity, and Egger’s and Begg’s tests were used to evaluate publication bias.

Semaglutide showed the highest weight regain after discontinuation (MD = -5.15 kg; 95% CI: -5.27 to -5.03), followed by exenatide (MD = -3.06 kg; 95% CI: -3.91 to -2.22), liraglutide (MD = -1.50 kg; 95% CI: -2.41 to -0.26), and orlistat (MD = -1.66 kg; 95% CI: -2.75 to -0.58). Heterogeneity was moderate to high (I² ranging from 41.7% to 99.7%). Egger’s test showed significant bias for liraglutide (p = 0.013), while no major bias was found for the other agents.

This meta-analysis demonstrates that weight regain is common and drug-dependent following the discontinuation of anti-obesity pharmacotherapies. The findings emphasize the need for sustained, long-term treatment strategies to maintain weight loss and to manage obesity as a chronic disease.

## Introduction and background

Obesity is a chronic, relapsing, and multifactorial disease, associated with increased morbidity, mortality, and healthcare burden globally [1]. Despite lifestyle interventions being the first-line treatment, their long-term effectiveness remains limited, and pharmacotherapy has become an essential adjunct in managing obesity [2]. In recent years, glucagon-like peptide-1 (GLP-1) receptor agonists, such as semaglutide, liraglutide, and exenatide, have emerged as highly effective agents for inducing substantial weight loss [3]. However, a growing body of evidence indicates that discontinuation of these agents often leads to weight regain, posing a challenge to long-term obesity management [4,5].

Weight regain following peak weight loss is believed to result from complex biological adaptations, including reduced resting energy expenditure and increased activity of appetite-regulating hormones [6,7]. This rebound effect is not unique to GLP-1 receptor agonists, but has also been observed with other pharmacotherapies, such as orlistat and phentermine [8,9]. Understanding the magnitude and consistency of this phenomenon across different drug classes is crucial for guiding clinical decisions, setting patient expectations, and informing long-term treatment strategies [10].

Despite the effectiveness of anti-obesity drugs in inducing initial weight loss, weight regain following treatment discontinuation remains a major clinical challenge, undermining long-term outcomes [11]. This rebound effect is driven by complex physiological adaptations, such as increased appetite, reduced energy expenditure, and hormonal changes that favor weight restoration [12,13].

The need to study this phenomenon is particularly crucial for agents like semaglutide and liraglutide, which have demonstrated profound weight loss but also significant regain after cessation, as observed in the STEP 4 and SCALE trials [13,14]. Weight regain after weight loss is a multifactorial process driven by adaptive physiological responses - such as increased ghrelin, reduced leptin, lower peptide YY levels, and decreased energy expenditure - that promote appetite and energy conservation.

This review focuses on semaglutide, liraglutide, exenatide, and orlistat, which represent distinct, well-established anti-obesity mechanisms (GLP-1 receptor agonism and lipase inhibition) with robust randomized evidence and long-term regulatory approval. This selection allows for a comprehensive comparison of their efficacy, durability, and post-treatment rebound patterns across pharmacologic classes.

Analyzing these four drugs offers essential insights into weight recidivism and supports the development of more effective, long-term obesity management strategies. This study aimed to quantify the magnitude of weight regained from the lowest weight achieved in individuals treated with anti-obesity drugs, focusing on semaglutide, liraglutide, exenatide, and orlistat. By analyzing data from randomized controlled trials (RCTs), it assessed weight regain during ongoing therapy and after treatment discontinuation, highlighting the clinical impact of rebound weight on long-term obesity care.

Key objectives included comparing weight regain across drug classes and evaluating dose- and duration-dependent effects. The findings aim to guide clinicians and policymakers in making informed decisions regarding the need for sustained pharmacologic and lifestyle interventions to support long-term weight maintenance in obesity care.

## Review

Methods

This study was conducted in accordance with the Preferred Reporting Items for Systematic Reviews and Meta-Analyses (PRISMA) guidelines [15], for conducting systematic reviews and meta-analyses and was registered with PROSPERO (registration number: CRD420251083591).

Search Strategy

A comprehensive literature search was independently conducted by two reviewers across five major electronic databases - PubMed, Scopus, Web of Science, ScienceDirect, and the Cochrane Library - to identify eligible RCTs evaluating weight loss, weight regain, and rebound effects associated with anti-obesity pharmacotherapies. The search covered studies published from January 2010 to October 2024, a period selected to capture recent advances in obesity pharmacotherapy, particularly with newer agents such as GLP-1 receptor agonists and dual agonists. A full list of search terms and Boolean operators used in each database is presented in Table 1. To ensure the comprehensiveness of the search, the reference lists of all eligible articles and relevant systematic reviews were also manually screened for additional studies. No language restrictions were applied. Identified articles were imported into EndNote version 20.2.1 (Clarivate Analytics, Philadelphia, PA, USA) for duplicate removal. These databases were selected for their extensive indexing of peer-reviewed clinical and pharmacological studies relevant to obesity treatment.

**Table 1:** Search strategy

Database	Search Count	Search Terms (With Boolean Operators)
PubMed	498	("weight loss" OR "body weight reduction" OR "weight regain" OR "rebound weight gain") AND ("anti-obesity drugs" OR "GLP-1 receptor agonists" OR "semaglutide" OR "liraglutide" OR "orlistat" OR "tirzepatide") AND ("randomized controlled trial" OR "RCT")
Scopus	258	TITLE-ABS-KEY("weight loss" AND "anti-obesity" AND ("semaglutide" OR "liraglutide" OR "orlistat" OR "exenatide" OR "tirzepatide") AND "randomized controlled trial")
Web of Science	398	TS=("weight regain" OR "body weight maintenance") AND TS=("pharmacotherapy" OR "anti-obesity drugs" OR "semaglutide" OR "liraglutide") AND TS=("randomized controlled trial")
ScienceDirect	248	("weight loss" AND "anti-obesity medication" AND ("GLP-1" OR "semaglutide" OR "liraglutide")) AND ("RCT" OR "randomized controlled")
Cochrane Library	41	("obesity" AND "drug therapy" AND "weight regain") in Trials database

Study Selection

The study selection for this meta-analysis was guided by a PICO framework, with a central focus on weight regain. The population comprised overweight or obese individuals enrolled in RCTs. The intervention included pharmacological anti-obesity treatments such as GLP-1 receptor agonists (e.g., semaglutide, liraglutide, and exenatide) and other agents, like orlistat or phentermine. The comparator varied across studies and included placebo, lifestyle modifications, or alternative drug regimens. The primary outcome was weight, in kilograms, regained from the lowest weight achieved - defined as the increase in body weight after reaching the nadir (minimum) mean body weight during treatment, typically representing the point of maximum therapeutic effect. Studies were included if they reported both the peak weight loss and the final weight at study endpoint or post-treatment follow-up, enabling calculation of the weight regained during or after discontinuation of therapy. Only RCTs with at least 12 weeks of treatment or follow-up duration were eligible. Studies were excluded if they lacked quantitative data on weight regain, were not pharmacologically focused, involved surgical or behavioral interventions alone, or used animal models. Non-randomized or open-label studies were only included if they provided clearly extractable data on peak weight loss and subsequent regain.

Data Extraction

Two reviewers independently assessed the papers on EndNote 20.2.1, based on their titles and abstracts, in accordance with the qualifying requirements. EndNote 20.2.1 was chosen for title and abstract screening due to its efficient reference management capabilities and automatic duplicate detection, which streamlined the initial study selection process. The full texts of the remaining papers were separately reviewed, and disagreements were resolved by a third, blinded reviewer, who independently made a final decision on disputed studies, ensuring an unbiased resolution process. Data extraction was carried out using a structured Excel spreadsheet (Microsoft® Corp., Redmond, WA, USA), which was pre-designed to capture essential information such as study design, sample size, population characteristics, intervention and comparator details, treatment duration, baseline and peak body weight, and weight at follow-up or post-discontinuation. Where numerical values for body weight at specific time points were not explicitly reported in the text or tables, the tool WebPlotDigitizer (v4.5) was employed to extract data from published graphs. This facilitated the accurate retrieval of weight measures at peak loss and end-of-study time points, which were critical for calculating weight regain.

To assess methodological quality and risk of bias, the Cochrane Risk of Bias 2.0 (RoB 2.0) tool was used [16]. This evaluation was independently performed by two reviewers, and discrepancies were resolved through consensus or consultation with a third reviewer. RoB 2.0 assessed five key domains: the randomization process, deviations from intended interventions, missing outcome data, measurement of the outcome, and selection of the reported result. Studies were categorized as having low risk, some concerns, or high risk of bias. The results were represented through traffic light plots and summary bar charts using the Cochrane Risk-of-Bias VISualization (Robvis) tool [17]. In the traffic light plot, green, yellow, and red indicators represented low, moderate, and high risk of bias, respectively, across domains. Expected findings included a low to moderate risk of bias in most domains, with occasional high bias in participant selection.

Statistical Analysis

Statistical analysis in this meta-analysis was performed using Comprehensive Meta-Analysis software version 3.7, which facilitated the computation and synthesis of effect sizes across included studies. The primary outcome was weight regain, measured as the mean difference (MD) in body weight between the time point of peak weight loss and the end-of-study or post-discontinuation follow-up. For each drug subgroup, pooled MDs and their corresponding 95% confidence intervals (CI) were calculated. Both fixed-effects and random-effects models were applied, depending on the level of heterogeneity observed across studies. Heterogeneity was assessed using the I² statistic, where values above 50% were interpreted as indicating moderate to high heterogeneity, alongside Cochrane’s Q test to evaluate the statistical significance of between-study variation.

In cases where weight data were not directly reported, estimates were extracted from graphical representations using WebPlotDigitizer (v4.5), to extract numerical data points from graphical figures by two independent reviewers, with mean values cross-verified to minimize digitization error (<5% deviation threshold). Sensitivity analyses were conducted by varying model assumptions and excluding studies with a high risk of bias, to test the stability of results. To assess potential publication bias, both Egger’s regression test and Begg’s rank correlation test were employed. Additionally, funnel plot asymmetry was visually inspected, and Duval and Tweedie’s Trim and Fill method was used to estimate the number of potentially missing studies and adjust the pooled effect accordingly.

Results

**Figure 1:**
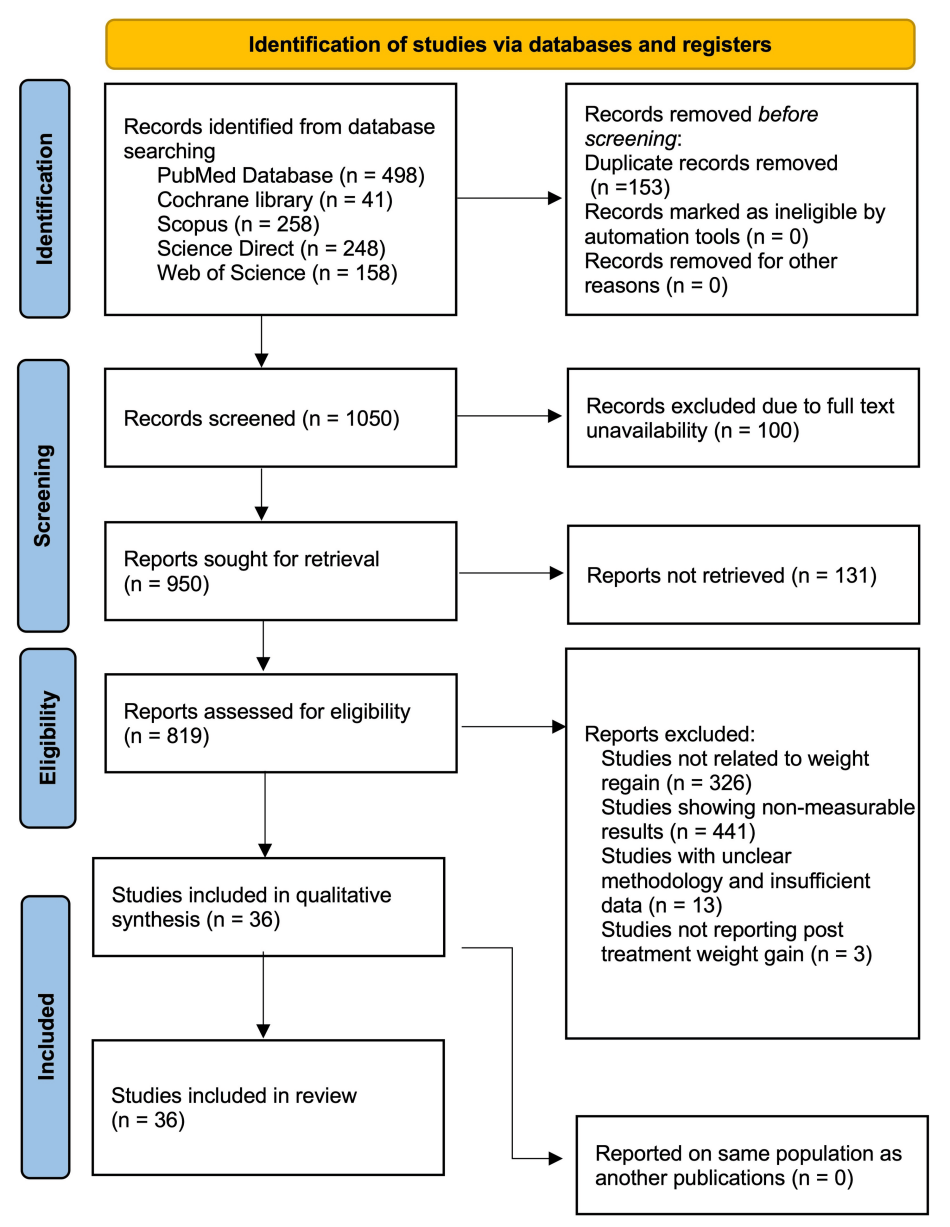
PRISMA flow chart

As depicted in the PRISMA flow diagram (Figure 1), a comprehensive search was conducted across five databases: PubMed (n = 498), Cochrane Library (n = 41), Scopus (n = 258), ScienceDirect (n = 248), and Web of Science (n = 158), yielding a total of 1,050 records after the removal of 153 duplicates. No records were excluded through automation tools or for other reasons at the initial stage. All 1,050 records were screened, and full-text retrieval was attempted, resulting in the elimination of 100 records. However, 131 reports could not be retrieved, yielding 819 studies. After eligibility assessment, 783 studies were excluded based on criteria such as irrelevance to weight regain (n = 326), non-measurable outcomes (n = 441), insufficient data (n = 13), and lack of post-treatment weight gain reporting (n = 3). Ultimately, 36 studies were included in the meta-analysis, contributing to the qualitative synthesis. No duplicate populations were identified across the included studies.

The included studies, as demonstrated in Table 2, comprise a diverse body of RCTs, open-label trials, prospective cohorts, and retrospective observational studies, with sample sizes ranging from small pilot studies (n = 16-56) to large multicenter phase 3 trials exceeding 2,000 participants. Most studies enrolled overweight or obese adults (BMI ≥ 27-43 kg/m²), with subgroups including individuals with metabolic risk factors, type 2 diabetes, non-alcoholic fatty liver disease (NAFLD), or post-bariatric weight regain. Interventions primarily evaluated anti-obesity pharmacotherapies such as orlistat, phentermine, sibutramine, liraglutide, semaglutide, tirzepatide, and exenatide, often in combination with lifestyle modification (diet, exercise, or cognitive-behavioral therapy). Treatment durations ranged from short-term 12-week interventions to long-term follow-up of up to three years, with some including off-treatment extensions to assess weight maintenance and regain. Across trials, active pharmacological treatments consistently achieved greater initial weight loss compared to placebo or lifestyle modification alone, though durability varied. GLP-1 receptor agonists (liraglutide, semaglutide, and exenatide) and the dual agonist tirzepatide demonstrated robust and clinically significant weight reductions, often exceeding 10%-15% of baseline body weight, while orlistat provided more modest but sustained benefits in weight maintenance. Key predictors of long-term outcomes included adherence, early weight loss response, lifestyle behaviors (dietary composition, sleep, and activity), and continuation of pharmacotherapy. Collectively, the evidence highlights obesity as a chronic, relapsing condition, with discontinuation of therapy frequently leading to partial weight regain and reversal of metabolic benefits. 

**Table 2:** Characteristics of the included studies VLED, Very-Low-Energy Diet; RCT, Randomized Controlled Trial; CV, Cardiovascular; WL, Weight Loss; BMI, Body Mass Index; LCD, Low-Calorie Diet; GI, Gastrointestinal; AE, Adverse Events; NAFLD, Non‐alcoholic Fatty Liver Disease; LFF, Low Fat Fraction; DE, Diet and Exercise; DB, Double-blinded; PC, Placebo-controlled; T2D, Type 2 Diabetes; BMI-SDS, Body Mass Index-Standard Deviation Score; OAD, Oral Antidiabetic Drug; ETD, Estimated Treatment Difference

Author (Year)	Study Design	Sample Size	Population	Interventions Used	Drug Dose(s)	Mean Age	Follow-Up/Duration	Diagnosis	Key Findings
Richelsen et al. (2007) [18]	RCT with 3-year follow-up after VLED	309 randomized (383 screened)	Obese adults (BMI ~37.5) with metabolic risk factors	8-week VLED (600-800 kcal/day) + 3 years orlistat 120 mg TID or placebo + lifestyle counseling	Orlistat 120 mg TID following 8-week VLED (600-800 kcal/day)	Not reported	3 years post-VLED randomized follow-up	Obese adults (BMI ~37.5) with metabolic risk factors	Orlistat group regained 2.4 kg less over 3 years (p = 0.02); 67% maintained ≥5% weight loss vs. 56% (p = 0.037); fewer developed type 2 diabetes (8 vs. 17 cases; p = 0.041); waist circumference reduced.
McVay et al. (2013) [19]	Randomized comparative intervention trial	144 (71 low-carb diet, 73 orlistat + low-fat diet)	Overweight/obese veterans (mean BMI 39.3 kg/m²; mean age 53 y)	Low-carb diet vs. orlistat 120 mg + low-fat diet; follow-up for 1 year	Orlistat 120 mg TID + low-fat diet vs low-carb diet	53 years	12 months	Overweight and obese veterans (BMI 39.3 kg/m²)	Pretreatment macronutrient intake did not predict final weight loss; higher pretreatment fat intake was linked with more rapid weight regain; macronutrient tailoring may slow regain but not improve final outcomes.
Marquez-Cruz et al. (2021) [20]	Prospective, open-label, multicenter study	932	Obese Mexican adults	Phentermine 15 mg or 30 mg once daily for 6 months	Phentermine 15 mg or 30 mg daily	Not reported	6 months	Obese Mexican adults	30 mg phentermine is more effective at 3 months but not 6; ~65% of 3-month responders maintained weight loss at 6 months; ~42% of non-responders lost ≥5% at 6 months; ~10% weight regain observed; no CV risk increase noted.
Madsen et al., 2008 [21]	3-year RCT with biomarker analysis	93	Obese adults (mean wt 108.9 ± 15.8 kg)	8-week VLED (800 kcal/day) + orlistat or placebo + lifestyle intervention for 3 years	Orlistat 120 mg TID vs placebo after 8-week VLED (800 kcal/day)	Not reported	3 years	Obese subjects (mean wt: 108.9 ± 15.8 kg)	Weight loss after VLED: 14.3 kg; after 3 years: 7.7 kg; orlistat group regained 3.9 kg less (p = 0.01); no group differences in biomarkers; adiponectin ↑ after VLED but returned to baseline unless >10% WL maintained over 3 years.
Wilding et al. (2022) [22]	RCT with off-treatment extension	327 (subset of 1961)	Adults with obesity (BMI ≥30 or ≥27 + comorbidity); no diabetes	Semaglutide 2.4 mg/week vs placebo for 68 weeks + lifestyle intervention; 52-week off-treatment follow-up	Semaglutide 2.4 mg weekly vs placebo + lifestyle	Not reported	68 weeks treatment + 52-week off-treatment follow-up	Obesity (BMI ≥30 or ≥27 with ≥1 comorbidity), no diabetes	Mean weight loss: 17.3% (semaglutide) vs 2.0% (placebo) at 68 weeks. After treatment withdrawal, ~2/3 of the weight was regained. Cardiometabolic benefits reversed, confirming obesity's chronic nature.
Sjöström et al. (1998) [23]	Double-blind, placebo-controlled RCT (2 years)	688 (out of 743)	Obese adults (BMI 28-47 kg/m²)	Orlistat 120 mg TID vs placebo; Year 1: hypocaloric diet; Year 2: weight maintenance (eucaloric) diet	Orlistat 120 mg TID vs placebo; Year 1 hypocaloric diet; Year 2 maintenance	Not reported	2 years	Obesity (BMI 28-47 kg/m²)	Greater weight loss with orlistat at 1 year (10.2% vs 6.1%). Continued orlistat use reduced weight regain. Cardiometabolic improvements noted. GI side effects are more common with orlistat.
Rössner et al. (2000) [24]	2-year multicenter RCT	Not explicitly reported	Obese adults (BMI 28-43 kg/m²)	Orlistat (60 or 120 mg TID) vs placebo; 1 year hypocaloric diet, 1 year weight maintenance diet	Orlistat 60 or 120 mg TID vs placebo	Not reported	2 years	Obesity (BMI 28-43 kg/m²)	Significant weight loss in both orlistat groups vs placebo at 1 year (up to 9.7%). Orlistat minimized weight regain in Year 2. Improved lipid profile, blood pressure, and quality of life. Mild GI AEs.
Rissanen et al. (2001) [25]	12-month double-blind RCT	51 women	Obese women (~44 y; BMI ~36.2 kg/m²)	Orlistat 120 mg TID vs placebo + hypoenergetic diet (600 kcal deficit); diet adjusted at 6 months	Orlistat 120 mg TID + hypoenergetic diet (600 kcal deficit)	~44 years	12 months	Obese women (BMI ~36.2 kg/m²)	Weight loss average 10 kg at 12 months. Orlistat had no independent effect on hemostatic markers. Sustained weight loss is linked to long-term reduction in PAI-1 and FVII activities. Fibrinogen unaffected.
Khoo et al. (2019) [26]	Randomized controlled trial (RCT)	30 (15 per group)	Obese adults with NAFLD (MRI LFF > 5%)	Liraglutide 3 mg daily vs. diet + exercise (DE) for 26 weeks, followed by 26-week observation (no active treatment)	Liraglutide 3.0 mg daily vs diet + exercise	40.7 ± 9.1 yrs	26 weeks treatment + 26-week observation	Obese adults with NAFLD (MRI LFF >5%)	Both groups achieved similar weight loss and LFF reduction at 26 weeks. Liraglutide group regained weight and LFF after discontinuation, while DE group maintained benefits. Lifestyle modification showed a more sustained effect.
Aronne et al. (2024) [27]	Phase 3 randomized withdrawal trial (SURMOUNT-4)	783 enrolled; 670 randomized	Adults (BMI ≥30 or ≥27 + comorbidity; mean 48 y)	Tirzepatide 10/15 mg weekly for 36 weeks, then randomized to continue tirzepatide or switch to placebo for 52 weeks	Tirzepatide 10/15 mg weekly; withdrawal vs continuation phase	48 yrs (mean)	36 weeks treatment + 52-week withdrawal	Obesity/overweight (BMI ≥30 or ≥27 with comorbidity)	Continued tirzepatide led to sustained and enhanced weight loss (25.3% total). Placebo group regained 14% weight. 89.5% in tirzepatide arm maintained ≥80% of initial weight loss vs 16.6% in placebo. GI AEs common but mild/moderate.
Gursoy et al. (2006) [28]	Prospective comparative study	182 (Orlistat: 98; Sibutramine: 84)	Obese adults (Orlistat n = 98; Sibutramine n = 84)	Orlistat vs. Sibutramine, both with diet and exercise prescriptions	Orlistat 120 mg TID vs Sibutramine dose per protocol + diet/exercise	Not reported	Multi-month program (follow-up ≈ 1 year)	Obese patients	Mean weight loss: 7.6% (orlistat), 10.5% (sibutramine). Sibutramine superior (p < 0.05). Compliance (56%) improved with early weight loss and physical activity. Noncompliance led to weight regain (~5-6 kg); no difference between drugs.
Lundgren et al. (2021) [29]	RCT (4-arm, head-to-head trial)	195	Obese adults (BMI 32-43), no diabetes	8-week LCD followed by 1 year of: (1) exercise + placebo, (2) liraglutide, (3) exercise + liraglutide, (4) placebo	Liraglutide 3.0 mg ± exercise vs placebo after 8-week LCD	Not stated	1 year	Obesity (BMI 32-43), no diabetes	Combination of liraglutide and exercise resulted in greatest weight and fat loss, and improved metabolic outcomes. Liraglutide alone was more effective than exercise alone. Benefits diminished with placebo.
Devlin et al. (2000) [30]	Open-label clinical trial with follow-up	16 obese women	Obese women with binge eating disorder (n = 16)	Phentermine + fluoxetine + cognitive-behavioral therapy (CBT); monthly maintenance offered for 3 years	Phentermine + Fluoxetine + CBT maintenance	Not stated	Up to 3 years (major weight regain after 1 year off therapy)	Obese women with binge eating disorder (BED)	Effective reduction in binge eating and weight during active treatment, but most weight was regained within 1 year post-treatment. Maintenance helped sustain binge reduction but not weight.
Kwon et al. (2022) [31]	RCT (sterol metabolism and weight change)	51 (Orlistat + Phen: 24; Placebo + Phen: 27)	Overweight/obese adults (BMI ≥ 27)	Orlistat 120 mg TID + phentermine 37.5 mg daily vs placebo + phentermine for 12 weeks	Orlistat 120 mg TID + Phentermine 37.5 mg daily vs placebo + Phentermine	Not reported	12 weeks active + 6-month follow-up	Overweight and obese adults (BMI ≥27)	Orlistat + Phen significantly reduced serum sterols and oxysterols. Improvements were reversed with weight regain at 6-month follow-up. Suggests orlistat may modulate endothelial dysfunction via sterol pathways.
Hill et al. (1999) [32]	Double-blind, placebo-controlled multicenter RCT	729 (from 1313 screened)	Obese adults (BMI 28-43) after ≥8% diet loss	After 6-month diet-induced weight loss (≥8%), randomized to orlistat (30, 60, or 120 mg TID) or placebo for 1 year	Orlistat 30/60/120 mg TID vs placebo	Not reported	1 year maintenance after the diet	Obese subjects with BMI 28-43	Orlistat 120 mg group regained significantly less weight (32.8%) vs placebo (58.7%); improved LDL and total cholesterol; more patients maintained ≥75% of weight loss. Demonstrated effectiveness in long-term maintenance.
Andersen et al. (2022) [33]	Substudy of RCT (S-LITE trial)	56 men	Obese men (BMI 32-43)	8-week 800 kcal/day diet; 1-year follow-up with: placebo, exercise, liraglutide, or combination	Liraglutide 3.0 mg ± exercise vs placebo after an 8-week 800 kcal diet	Not stated	1 year	Obese men (BMI 32-43)	Weight loss improved sperm concentration and count. Benefits were maintained at 1 year only in men who maintained the weight loss. No change in motility or semen volume.
Bogh et al. (2023) [34]	RCT; 2 × 2 factorial trial	195 adults	Obese adults (2 × 2 factorial design)	8-week LCD → 1-year maintenance: placebo or liraglutide ± exercise	Liraglutide 3.0 mg ± exercise vs placebo after LCD	Not stated	1 year	Obese adults	Poor sleep duration/quality predicted weight regain. Liraglutide increased sleep duration at 26 weeks; exercise preserved sleep quality improvements. Sleep is a predictor of long-term weight outcomes.
Jensen et al. (2022) [35]	RCT, double-blind, 1:1:1:1 randomization	130 (completers)	Obese adults post-weight-loss (n = 130 completers)	1-year: placebo, exercise, liraglutide, or both after LCD	Liraglutide vs exercise vs combination vs placebo	Stratified <40/≥40	1 year	Obese adults after weight loss	Liraglutide and exercise reduced appetite and sedentary time. Combination improved cognitive restraint and activity. Behavioral changes may contribute to long-term weight loss maintenance.
Jensen et al. (2023) [36]	Retrospective observational study	50 (29 liraglutide, 21 semaglutide)	Post-bariatric patients with weight regain	GLP1-RA (liraglutide/semaglutide) for 6 months in post-bariatric surgery patients	Liraglutide (1.8-3 mg) or Semaglutide (per protocol)	Not stated	6 months	Post-bariatric patients with weight regain	GLP1-RA reduced ~67% of regained weight post-bariatric surgery. Safe and effective. Supports GLP1-RA as a treatment option for post-surgery weight regain.
Davidson et al. (1999) [37]	Multicenter RCT, placebo-controlled, 2-year duration	892 randomized (223 placebo, 657 orlistat)	Obese adults (BMI 30-43)	Orlistat 120 mg or 60 mg TID vs placebo + controlled-energy diet for 2 years	Orlistat 60 or 120 mg TID vs placebo + controlled-energy diet	Not reported	2 years	Obese adults (BMI 30-43)	Orlistat led to greater weight loss and less weight regain vs placebo. Improved LDL cholesterol and insulin. Long-term orlistat treatment was effective for sustained weight management and metabolic improvements.
Le Roux et al. (2017) [38]	RCT, DB, PC	2254	Obesity + Prediabetes (n = 2254)	Liraglutide 3.0 mg vs Placebo	Liraglutide 3.0 mg daily vs placebo	NA	160 weeks (3 years)	Obesity + Prediabetes	Liraglutide delayed the onset of diabetes (HR = 0.21); greater weight loss over 160 weeks.
Rubino et al. (2021) [39]	RCT, DB	803	Overweight/obesity (no diabetes)	Semaglutide 2.4 mg vs Placebo	Semaglutide 2.4 mg weekly vs placebo	46	68 weeks	Overweight/Obesity, No DM	Continued semaglutide led to -14.8% weight difference over 48 weeks vs placebo.
Kelly et al. (2020) [40]	RCT, DB	251	Adolescents with obesity (n = 251)	Liraglutide 3.0 mg + lifestyle vs Placebo + lifestyle	Liraglutide 3.0 mg daily + lifestyle vs placebo + lifestyle	12-18	12-18 months	Adolescent Obesity	Greater BMI-SDS reduction; 5-10% BMI reduction is more frequent in the liraglutide group.
Davies et al. (2015) [41]	RCT, DB	846	Obesity + Type 2 Diabetes (n = 846)	Liraglutide 3.0 mg, 1.8 mg vs Placebo	Liraglutide 3.0 mg or 1.8 mg vs placebo	NA	52 weeks	Obesity + Type 2 DM	Liraglutide 3.0 mg led to 6% weight loss vs 2% in placebo; improved glycemic control.
Kendall et al. (2005) [42]	30-week, double-blind, placebo-controlled RCT	733	T2D uncontrolled on Met + SU (n = 733)	Exenatide 5 µg or 10 µg subcutaneous BID + metformin + sulfonylurea vs placebo	Exenatide 5 µg or 10 µg BID subcutaneous	55 ± 10 yrs	30 weeks	Type 2 Diabetes uncontrolled on MET + SU	Exenatide significantly reduced HbA1c (-0.8% to -1.0%) vs placebo, with associated weight loss (-1.6 kg), and higher % achieving HbA1c ≤7%. Well tolerated with mild/moderate nausea and hypoglycemia in 19-28% of patients.
Guja et al. (2018) [43]	28-week, multicenter, double-blind RCT	464	T2D on insulin glargine + Met (n = 464)	Exenatide QW 2 mg + insulin glargine vs placebo + insulin glargine	Exenatide QW 2 mg + insulin glargine vs placebo	58 yrs	28 weeks	Type 2 Diabetes on basal insulin + MET	Exenatide QW improved HbA1c (-0.73%), reduced weight (-1.5 kg), improved postprandial glucose, and more patients reached HbA1c <7%. GI and injection-site AEs were higher; hypoglycemia was similar in both groups.
Gadde et al. (2017) [44]	28-week, open-label, multicenter, comparative trial	365	T2D on Metformin (n = 365)	Exenatide QWS-AI 2.0 mg weekly vs sitagliptin 100 mg daily vs placebo	Exenatide QWS-AI 2 mg weekly vs Sitagliptin 100 mg vs Placebo	Not reported	28 weeks	Type 2 Diabetes on metformin monotherapy	Exenatide QWS-AI reduced HbA1c more than sitagliptin or placebo (-1.13% vs -0.75% and -0.40%), with weight loss (~-1.1 kg) and better HbA1c target achievement. GI and injection-site AEs are common, but no new safety signals.
Herold et al. (2020) [45]	24-week randomized, placebo-controlled trial + 6-mo FU	79	Type 1 Diabetes (with/without C-peptide)	Exenatide ER 2 mg weekly vs placebo, stratified by C-peptide positivity	Exenatide ER 2 mg weekly vs placebo	Not reported	24 weeks + 6-month follow-up	Type 1 Diabetes with/without C-peptide	Modest HbA1c reduction at 12 weeks (p = 0.01), not sustained at 24 weeks (p = 0.08). Greater benefit in the C-peptide + subgroup. Weight loss observed; no increase in hypoglycemia. More AEs in the exenatide ER group.
Kaku et al. (2018) [46]	Phase III, open-label, 56-week trial	601	Japanese T2D adults (n = 601)	Semaglutide 0.5 mg or 1.0 mg weekly vs one additional oral antidiabetic drug (OAD)	Semaglutide 0.5 mg or 1.0 mg weekly vs OAD	Not reported (Japanese adults)	56 weeks	Type 2 Diabetes inadequately controlled on diet/OAD	Semaglutide 0.5 mg and 1.0 mg significantly reduced HbA1c (-1.7% and -2.0%) and body weight (-1.4 kg and -3.2 kg) compared to additional OAD. >80% achieved HbA1c <7%. GI adverse events were most frequent but generally mild and transient.
Davies et al. (2021) [47]	Phase III, double-blind, double-dummy RCT (68 weeks)	1210	Overweight/obese T2D adults (STEP-2)	Semaglutide 2.4 mg, 1.0 mg, or placebo weekly, all with lifestyle intervention	Semaglutide 2.4 mg or 1.0 mg weekly vs placebo	Not reported	68 weeks	Overweight/obese adults with Type 2 Diabetes	Semaglutide 2.4 mg significantly reduced body weight (-9.6%) compared to placebo (-3.4%). 68.8% achieved ≥5% weight loss vs 28.5% with placebo. GI adverse events were more frequent but mostly mild/moderate. Superior efficacy observed vs 1.0 mg.
Wharton et al. (2023) [48]	STEP 5 Trial, 104-week RCT with lifestyle intervention	174 (subset for eating control analysis)	Adults with obesity (subset n = 174)	Semaglutide 2.4 mg weekly vs placebo + lifestyle intervention	Semaglutide 2.4 mg weekly vs placebo + lifestyle	Not reported	104 weeks (2 years)	Overweight/obese adults	Semaglutide 2.4 mg led to -14.8% weight loss vs -2.4% with placebo at 104 weeks. Significant improvements in craving control, hunger, fullness, and mood were observed across multiple domains. Weight reduction correlated with improved eating behavior scores.
Pratley et al. (2019) [49]	Phase 3a, randomized, double-blind, double-dummy trial (52 weeks)	711	T2D on Met ± SGLT2i (n = 711)	Oral semaglutide 14 mg vs subcutaneous liraglutide 1.8 mg vs placebo (all once daily)	Oral Semaglutide 14 mg daily vs Liraglutide 1.8 mg vs Placebo	56 yrs	52 weeks	Type 2 Diabetes (on metformin ± SGLT2i)	Oral semaglutide was non-inferior to liraglutide and superior to placebo in reducing HbA1c (-1.2%) and weight (-4.4 kg). GI events were more frequent but mostly mild.
Yamada et al. (2020) [50]	52-week phase 2/3a randomized controlled trial in Japan	243	Japanese T2D patients (n = 243)	Oral semaglutide 3, 7, or 14 mg vs placebo vs liraglutide 0.9 mg (subcut)	Oral Semaglutide 3, 7, 14 mg vs Placebo vs Liraglutide 0.9 mg	Not reported	52 weeks	Japanese patients with T2D (diet or OAD-managed)	Oral semaglutide showed dose-dependent HbA1c reductions (up to -1.7%) superior to placebo and comparable to liraglutide. GI events were most common but mild; constipation most frequent.
Husain et al. (2019) [51]	PIONEER 6: RCT, double-blind, placebo-controlled (CV outcomes)	3183	T2D with high CV risk (n = 3183)	Oral semaglutide 14 mg daily vs placebo	Oral Semaglutide 14 mg daily vs Placebo	66 yrs	Median 15 months (CV outcomes trial)	T2D with high CV risk	Oral semaglutide showed non-inferior CV safety (HR = 0.79 for major CV events), reduced CV deaths (HR = 0.49), and all-cause mortality (HR = 0.51). GI events led to more treatment discontinuations.
Coelho et al. (2023) [52]	Pilot RCT; double-blind, placebo-controlled (GLIDE trial)	27	Post-LAGB obese T2D adults (n = 27)	LAGB + liraglutide 1.8 mg vs LAGB + placebo (6 months + 6-month follow-up)	LAGB + Liraglutide 1.8 mg vs LAGB + Placebo	Not reported	6 months treatment + 6-month follow-up	T2DM and obesity post-LAGB	No significant difference in HbA1c or weight at 6 months. At 12 months, both HbA1c and body weight were significantly higher in the liraglutide group. Liraglutide offered no additional benefit post-LAGB. The trial was underpowered.
McGowan et al. (2024) [53]	Phase 3 RCT, double-blind, parallel-group (STEP 10)	207	Adults with obesity + prediabetes (n = 207)	Semaglutide 2.4 mg weekly vs placebo + diet and exercise (52 weeks)	Semaglutide 2.4 mg weekly vs Placebo + Diet/Exercise	53 yrs	52 weeks	Obesity and prediabetes	Semaglutide resulted in -13.9% weight loss vs -2.7% with placebo (ETD: -11.2%, p < 0.0001). 81% reverted to normoglycemia vs 14% on placebo. Safety profile consistent with GLP-1 RA class; no new safety signals detected.

Risk of Bias

**Figure 2:**
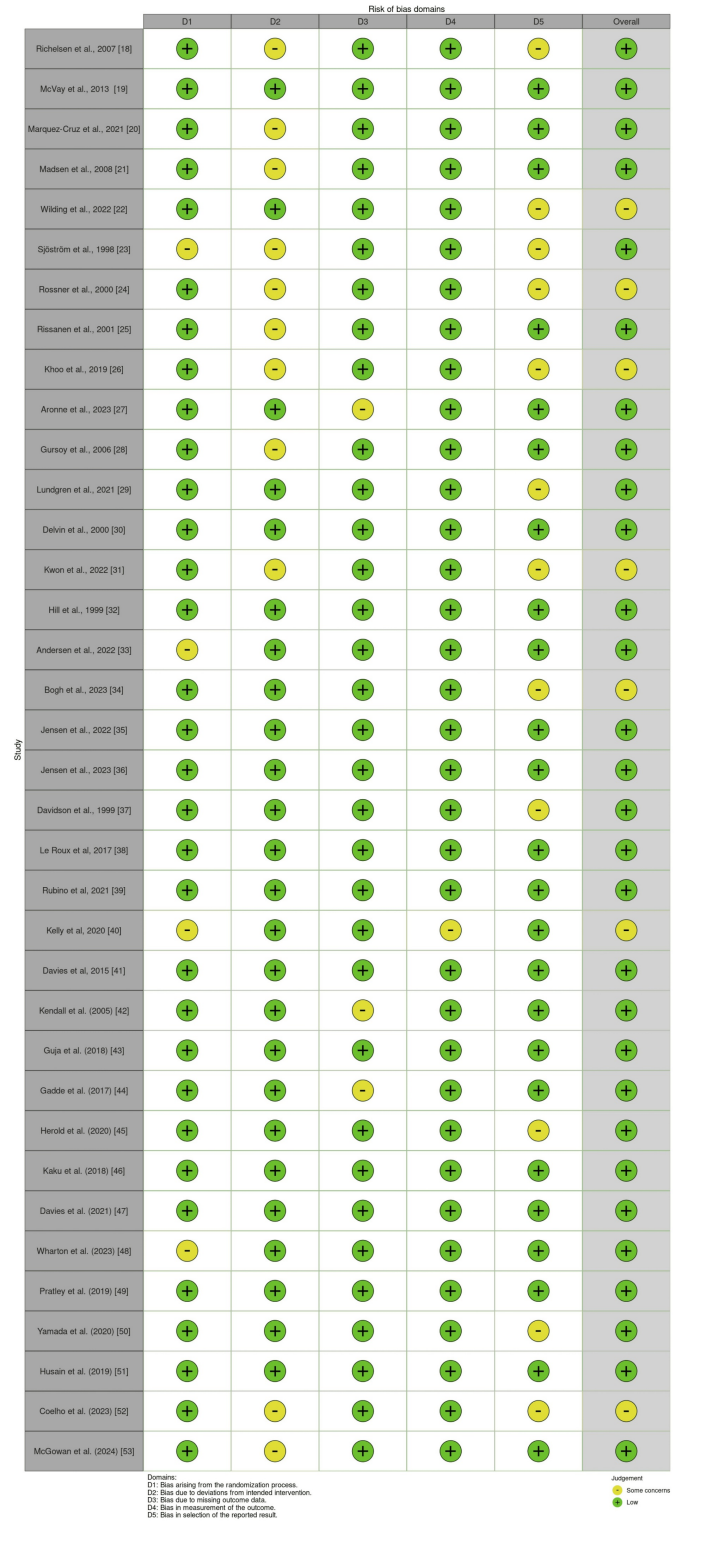
Risk of bias assessment for the included studies

The RoB 2 assessment, as shown in Figure 2, across the included RCTs, demonstrated a generally low risk of bias, with several studies exhibiting “some concerns” in specific domains. Most studies showed low risk in the randomization process (D1), reflecting adequate random sequence generation and allocation concealment. However, a few studies, such as those by Sjöström et al. (1998) [23], Andersen et al. (2022) [33], and Kelly et al. (2020) [40], showed some concerns due to incomplete reporting of randomization or stratification procedures. Deviations from intended interventions (D2) raised some concerns in studies such as McVay et al. (2015) [19], Marquez-Cruz et al. (2021) [20], and Coelho et al. (2023) [52], primarily due to open-label designs or unblinded participants, which could have influenced adherence or behavior. In contrast, studies with well-implemented blinding and adherence monitoring, such as Wilding et al. (2022) [22] and Davies et al. (2021) [47], were rated low risk. Regarding missing outcome data (D3), the majority of studies maintained good follow-up rates. However, Coelho et al. (2023) [52] and Kwon et al. (2022) [31] had some concerns due to small sample sizes and notable attrition, increasing the potential for bias. Measurement of the outcome (D4) was largely at low risk, as most trials used objective measures (e.g., body weight and HbA1c). Bias in the selection of reported results (D5) was typically low, except in a few trials where protocol deviations or selective reporting could not be ruled out. Overall, the majority of studies were judged to be at low risk of bias, reinforcing the reliability of their findings. However, studies with “some concerns” should be interpreted with caution in meta-analytic synthesis.

On-Treatment Erosion

**Figure 3:**
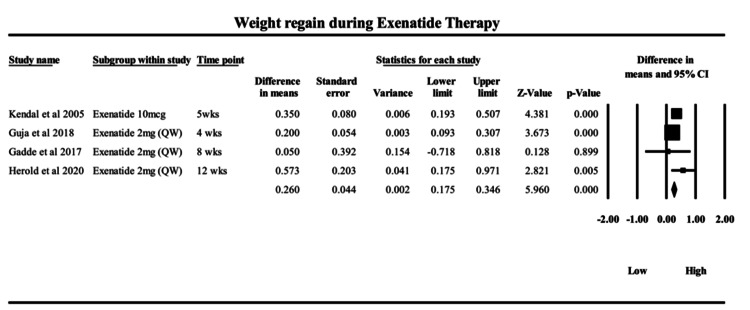
Forrest plot on the weight regain following exenatide therapy from 4 weeks to 12 weeks Forest plot showing mean differences in weight regain across randomized controlled trials of exenatide. Studies included: Kendall et al. (2005) [42]; Guja et al. (2018) [43]; Gadde et al. (2017) [44]; and Herold et al. (2020) [45]. Diamonds represent pooled estimates, and squares represent individual study effects with 95% confidence intervals.

Exenatide: The meta-analysis, as presented in Figure 3, evaluating weight regain during ongoing exenatide therapy - specifically the difference between peak weight loss from baseline and the final end-of-study weight loss - revealed a pooled MD of 0.26 kg (95% CI: 0.17 to 0.35; p < 0.00001) when compared to placebo. This small but statistically significant regain indicates that, despite continued administration of exenatide, a modest rebound in weight may occur, potentially reflecting a pharmacological tolerance effect over time. The fixed-effect model, applied due to low heterogeneity (I² = 41.7%, p = 0.16), suggests the results are relatively consistent across studies. The random-effects model yielded a slightly higher MD of 0.29 kg (95% CI: 0.15 to 0.44), reinforcing the robustness of the finding. Among the included studies, Herold et al. (2020) [45] contributed the largest weight to the pooled estimate due to its longer duration (12 weeks) and higher event rate (MD = 0.573), suggesting that the degree of rebound may correlate with the duration of therapy. Shorter trials, such as Kendall et al. (2005) [42] and Guja et al. (2018) [43], demonstrated less regain, while Gadde et al. (2017) [44] showed minimal change - emphasizing a potential dose- and duration-dependent waning effect. Exenatide, a short-acting GLP-1 receptor agonist, primarily exerts its effect by enhancing glucose-dependent insulin secretion, slowing gastric emptying, and reducing appetite through central satiety pathways. However, its shorter half-life and intermittent receptor engagement may lead to quicker attenuation of anorectic effects, explaining the modest on-treatment kilograms regained from the lowest weight achieved and the tendency for early plateauing in weight loss, despite continued therapy. Clinically, while the observed weight regain is relatively minor in absolute terms, it highlights a critical consideration for long-term exenatide therapy: even in the absence of treatment discontinuation, patients may exhibit a plateau or reversal in weight loss benefits. This may necessitate complementary behavioral interventions or combination pharmacotherapy in longer-term regimens. The findings underscore the importance of monitoring patient weight trajectories throughout treatment to optimize therapeutic outcomes.

**Figure 4:**
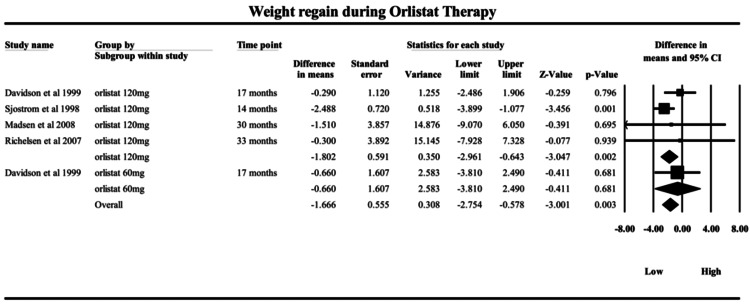
Forrest plot on the weight regain during orlistat therapy from 14 months to 33 months Forest plot showing mean differences in weight regain across randomized controlled trials of orlistat. Studies included: Davidson et al. (1999) [37]; Sjöström et al. (1998) [23]; Madsen et al. (2008) [21]; and Richelsen et al. (2007) [18]. Squares represent individual study estimates with 95% confidence intervals, and the diamond represents the pooled estimate.

Orlistat:* *The meta-analysis, as shown in Figure 4, evaluating weight regain during ongoing orlistat therapy - comparing peak weight loss (from baseline) to end-of-study weight - revealed an MD of -1.66 kg (95% CI: -2.75 to -0.58; p = 0.0027). This statistically significant result suggests that participants regained approximately 1.66 kg of the weight they had previously lost by the trial's end, even while still receiving orlistat. This reflects a partial erosion of therapeutic benefit over time, potentially indicating the development of tolerance or adaptation during extended use. Importantly, heterogeneity was low to absent (Q = 3.33, df = 4, p = 0.50; I² = 0%), suggesting that the observed effect was consistent across studies and unaffected by inter-study variability. The fixed-effects model was, therefore, appropriate. Among the included trials, Sjöström et al. (1998) [23] provided the strongest contribution, with an MD of -2.49 kg (95% CI: -3.90 to -1.08), both highly significant and precise. In contrast, Davidson et al. (1999) [37], which used a lower dose of 60 mg three times daily (vs. 120 mg in other studies), showed a smaller, nonsignificant regain (MD = -0.66 kg, p = 0.68), possibly indicating dose-dependent differences in sustained efficacy. Richelsen et al. (2007) [18] and Madsen et al. (2008) [21] had higher variability and wider CIs, likely due to longer study durations or population differences. Unlike centrally acting agents, orlistat functions peripherally by inhibiting gastrointestinal lipases, thereby reducing dietary fat absorption by approximately 30%. Because it lacks central appetite modulation, the modest on-treatment erosion in weight observed likely reflects behavioral and dietary adaptation, rather than pharmacologic tolerance. This mechanism also explains the consistent but smaller weight regained from the lowest weight achieved, compared to GLP-1 receptor agonists. Clinically, these findings are critical: even with continued therapy, some weight regain occurs after peak loss, emphasizing that pharmacologic intervention alone may be insufficient for long-term weight maintenance. The data support the idea that orlistat should be paired with ongoing dietary, behavioral, and physical activity support. Furthermore, the observed dose-related differences suggest that higher orlistat doses (120 mg) may offer more durable effects. This evidence aids clinicians in setting realistic expectations for patients and reinforces the need for sustained lifestyle modification, even during active pharmacotherapy.

**Figure 5:**
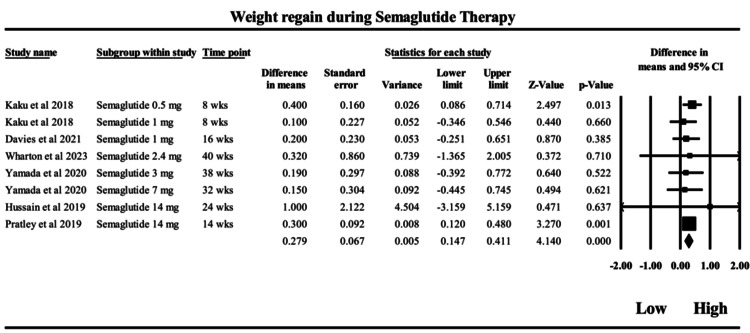
Forrest plot on the weight regain during semaglutide therapy from 8 weeks to 40 weeks Forest plot showing mean differences in weight regain across randomized controlled trials of semaglutide. Studies included: Kaku et al. (2018) [46]; Davies et al. (2021) [47]; Wharton et al. (2023) [48]; Yamada et al. (2020) [50]; Husain et al. (2019) [51]; and Pratley et al. [49]. Squares represent individual study estimates with 95% confidence intervals, and the diamond represents the pooled estimate.

Semaglutide: The meta-analysis, as depicted in Figure 5, evaluated the rebound effect in weight following peak weight loss among patients treated with semaglutide by comparing the difference between peak and end-of-trial weight loss from baseline. The overall MD was 0.279 kg (95% CI: 0.147 to 0.411; p < 0.001), indicating a modest but statistically significant weight regain from the point of maximal weight reduction to the end of treatment. This value reflects a small rebound effect, suggesting that while semaglutide is effective in promoting weight loss, there is a tendency for some degree of regain over time during continued treatment. Importantly, heterogeneity was negligible, as evidenced by an I² value of 0% and a non-significant Q-test (Q = 1.75, df = 7, p = 0.972). This implies consistent findings across the included studies, regardless of variations in dose or duration, lending robustness to the pooled estimate. The fixed- and random-effects models yielded identical estimates, further supporting the consistency of the data. Semaglutide is a long-acting GLP-1 receptor agonist with prolonged receptor occupancy, leading to sustained appetite suppression and delayed gastric emptying. The minimal on-treatment weight regained from the lowest weight achieved may therefore represent gradual physiological adaptation, rather than a loss of pharmacologic potency. This is consistent with the drug’s extended duration of action and stable incretin effect, distinguishing it from shorter-acting analogs like exenatide. In terms of study contributions, Pratley et al. (2019) [49], with semaglutide 14 mg, showed a high weight regain (effect size = 0.3), and Kaku et al. (2018) [46], with semaglutide 0.5 mg, also reported substantial regain (effect size = 0.4). However, high-dose regimens such as semaglutide 14 mg showed greater variability in effect sizes [51], possibly due to differential adherence or metabolic adaptation. Clinically, these findings highlight that semaglutide demonstrates sustained weight loss with minimal rebound, even over long durations and varying doses. The small magnitude of regain observed should be interpreted in the context of the drug’s continued efficacy, suggesting that long-term maintenance strategies - possibly including behavioral or nutritional interventions - may help mitigate this rebound effect and improve overall weight management outcomes.

**Figure 6:**
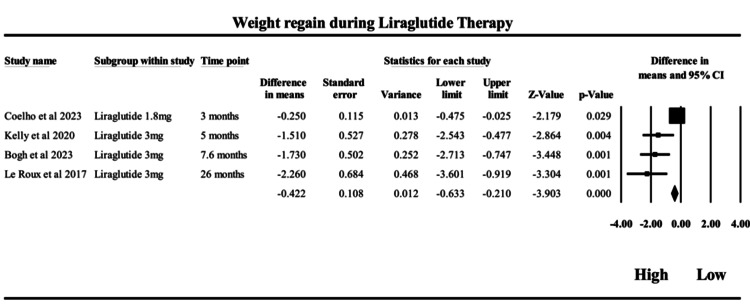
Forrest plot on the weight regain during liraglutide therapy from 3 months to 26 months Studies included: Coelho et al. (2023) [52]; Kelly et al. (2020) [40]; Bogh et al. (2023) [34]; and Le Roux et al. (2017) [38]. Squares represent effect sizes with 95% confidence intervals for each study, while the diamond represents the pooled estimate.

Liraglutide: The present meta-analysis, as presented in Figure 6, evaluated the kilograms regained from the lowest weight achieved in single-arm studies of liraglutide (1.8 mg and 3 mg) across four trials, with treatment durations ranging from 3 to 26 months. The pooled estimate using a fixed-effects model showed a statistically significant MD of -0.42 kg (95% CI: -0.63 to -0.21; p < 0.0001), indicating that, on average, patients regained only 0.42 kg after reaching peak weight loss. This suggests that liraglutide maintains a clinically durable weight loss effect over time. However, the random-effects model, which accounts for study heterogeneity, revealed a larger pooled MD of -1.34 kg (95% CI: -2.41 to -0.26; p = 0.015), further confirming the benefit while acknowledging greater variability between studies. The heterogeneity was substantial (Q = 20.52, p = 0.00013; I² = 85.4%), indicating variability likely attributable to differences in dose, study population, and particularly, treatment duration. Notably, Le Roux et al. (2017) [38], which followed patients for 26 months, contributed the most weight regain (MD = -2.26 kg), whereas Coelho et al. (2023) [52], with a shorter three-month duration, reported the least (MD = -0.25 kg). This trend suggests that tolerance to liraglutide may develop over time, diminishing its anorectic effect and contributing to gradual weight regain in the longer term. Liraglutide, an intermediate-acting GLP-1 receptor agonist, acts centrally to suppress appetite and peripherally to slow gastric emptying. The modest on-treatment kilograms regained from the lowest weight achieved reflect gradual attenuation of central satiety signaling with prolonged exposure. However, its steady receptor activation contributes to more durable weight maintenance compared to shorter-acting agents, aligning with its intermediate pharmacokinetic profile. Clinically, these findings highlight the effectiveness of liraglutide in sustaining weight loss with relatively modest regain. However, the observed trend of increased weight regain with longer duration emphasizes the need for long-term management strategies, including dose adjustments, behavioral reinforcement, or adjunct therapies to combat pharmacological tolerance and maintain treatment efficacy. These results support liraglutide’s role as a valuable component in chronic obesity management, but also call for vigilance against diminishing returns with extended use.

Post-discontinuation Regain

**Figure 7:**
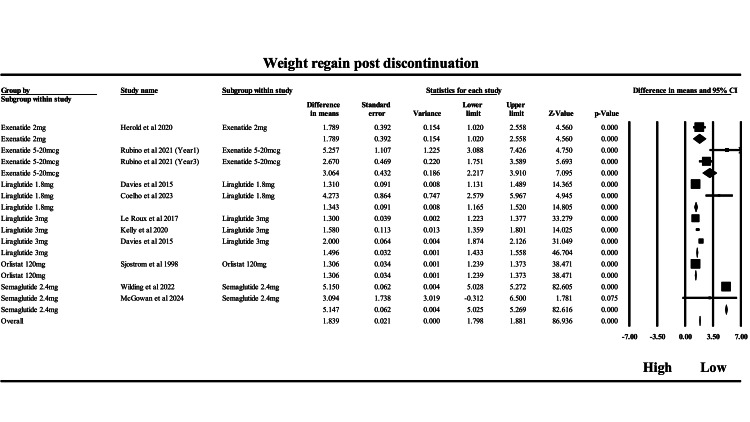
Forrest plot on the weight regain during post discontinuation period of anti-obesity drug therapy Forest plot showing mean differences in weight regain following discontinuation of exenatide, liraglutide, orlistat, and semaglutide therapies. Included studies: Herold et al. (2020) [45]; Rubino et al. (2021) [39]; Davies et al. (2015) [41]; Coelho et al. (2023) [52]; Le Roux et al. (2017) [38]; Kelly et al. (2020) [40]; Sjöström et al. (1998) [23]; Wilding et al. (2022) [22]; and McGowan et al. (2024) [53]. Squares represent individual study estimates with 95% confidence intervals, and the diamond represents the pooled estimate.

The results of this meta-analysis assessed weight regain over 12 weeks after discontinuation of anti-obesity pharmacotherapies, with data stratified by drug class and compared to placebo. As presented in Figure 7, the overall pooled MD was -1.84 kg (95% CI: -1.88 to -1.80; p < 0.001), favoring active treatment at peak weight loss over post-discontinuation follow-up, indicating a clinically relevant weight regain across agents once therapy ceased. A negative MD reflects that participants regained weight compared to their peak loss while on treatment, and this meta-analytic estimate underscores the rebound effect seen after stopping pharmacotherapy. Semaglutide 2.4 mg showed the largest effect, with a pooled MD of -5.15 kg (95% CI: -5.27 to -5.03), derived primarily from Wilding et al. (2022) [22], which contributed substantially due to its precision and low variance. This suggests a notable rebound after stopping semaglutide, highlighting its powerful weight-lowering effect during treatment and the vulnerability to regain after cessation. Exenatide 5-20 mcg, drawn from Rubino et al. (2021) [39] at both Years 1 and 3, showed a significant pooled MD of -3.06 kg (95% CI: -3.91 to -2.22), confirming moderate regain. Orlistat and liraglutide 1.8 mg both had modest regains, with MDs of -1.31 and -1.34 kg, respectively. Liraglutide 3 mg had a pooled MD of -1.50 kg, supported by high-precision data from Le Roux et al. (2017) and Davies et al. (2015) [38,41]. The overall heterogeneity was extremely high (I² = 99.7%), suggesting substantial variation between studies, likely due to differences in drug mechanisms, duration of prior therapy, dosing, and population characteristics. Some subgroups, like liraglutide 1.8 mg, showed particularly high I² (>90%), while semaglutide had much lower heterogeneity, indicating consistency across those studies. This analysis reinforces that discontinuation of anti-obesity medications is frequently followed by significant weight regain, with the magnitude proportional to the initial weight loss effect of the drug. Agents like semaglutide and high-dose exenatide, which induce greater weight loss, also demonstrate greater rebounds. These findings highlight the chronic nature of obesity and suggest that long-term, or even indefinite, treatment may be necessary for sustained weight management. Clinicians should prepare patients for the possibility of weight regain post-therapy and consider integrating behavioral and lifestyle interventions during and after pharmacologic treatment to mitigate this rebound.

Collectively, these results indicate that the degree of kilograms regained from the lowest weight achieved during active treatment varies according to each agent’s mechanism of action, being smallest for long-acting GLP-1 agonists (semaglutide), moderate for intermediate-acting liraglutide, more noticeable for short-acting exenatide, and lowest, but peripheral, for orlistat. This mechanistic gradient highlights the importance of pharmacologic duration and site of action in determining long-term weight maintenance efficacy.

Publication Bias

The assessment of publication bias across the five analyzed outcomes - semaglutide, exenatide, liraglutide, orlistat, and post-discontinuation at 12 weeks - utilized multiple statistical methods, including Begg’s rank correlation test, Egger’s test of the intercept, Fail-safe N, and Duval and Tweedie’s Trim and Fill method, as provided in the Appendix (Supplementary Figures 8-12). For semaglutide, Begg’s test showed no evidence of bias (Kendall’s tau b = 0.03571, p = 0.90154), and Egger’s test also indicated no small-study effects (intercept = -0.18380, p = 0.58764). The funnel plot was symmetric, and the Trim and Fill analysis did not suggest missing studies. Exenatide similarly showed no significant publication bias, with Begg’s test (tau b = 0.16667, p = 0.73410) and Egger’s intercept (0.08629, p = 0.97573) both non-significant. No studies were added via Trim and Fill, confirming symmetry in the funnel plot. In contrast, liraglutide exhibited potential publication bias. While Begg’s test was marginal (tau b = 0.53333, p = 0.13285), Egger’s test revealed a significant intercept (2.03048, p = 0.01329), suggesting small-study effects. Trim and Fill imputed three missing studies, shifting the effect size downward and highlighting a likely overestimation of treatment effects due to bias. For orlistat, both Begg’s and Egger’s tests showed no significant bias (tau b = 0, p = 1.00000; intercept = 0.78964, p = 0.37836); however, Trim and Fill still imputed three studies, which led to a more negative effect estimate - indicating some concern despite the non-significant tests. Finally, for post-discontinuation weight regain (12 weeks), Begg’s and Egger’s tests did not detect bias (p = 0.53342 and 0.64131, respectively), and Trim and Fill detected no missing studies, supporting the robustness of the results.

Discussion

This meta-analysis demonstrates that, while GLP-1 receptor agonists and other anti-obesity agents are effective in inducing substantial initial weight reduction, patients commonly experience clinically relevant weight regain from the lowest weight achieved, both during ongoing treatment and following discontinuation. For instance, even with continued therapy, exenatide users exhibited an average weight regain of approximately 0.26-0.29 kg, and orlistat users regained about 1.66 kg, indicating a partial attenuation of therapeutic benefit over time. Rebound was most pronounced post-discontinuation, with semaglutide 2.4 mg showing an MD of approximately -5.15 kg and exenatide approximately -3.06 kg, reflecting substantial regression toward baseline weights within a few months after therapy cessation. These findings are consistent with outcomes from pivotal trials, such as STEP 1 [22], which documented significant weight regain within one year of stopping semaglutide, and align with broader evidence from systematic reviews underscoring the chronic and relapsing nature of obesity [12,54].

From a clinical standpoint, these findings highlight the necessity of a long-term, integrated approach to obesity management that combines sustained pharmacotherapy with behavioral and lifestyle interventions. Patients should be counseled that anti-obesity medications function similarly to other chronic disease treatments - discontinuation is likely to result in weight regain. To enhance the long-term efficacy of treatment, clinicians should emphasize maintenance strategies, including ongoing dietary management, regular physical activity, and continuous follow-up. These recommendations are consistent with expert guidelines [55] and longitudinal data, which collectively underscore that durable weight control often requires prolonged or repeated therapeutic engagement to address the complex biological and behavioral mechanisms driving weight recidivism.

The findings of this meta-analysis, which demonstrate both modest weight regain during continued pharmacotherapy and substantial rebound following treatment discontinuation, are well aligned with existing literature not directly included in the current synthesis. For instance, Wadden et al. (2021) [56], in a year-long real-world study of semaglutide, observed that while initial weight loss was substantial, individuals who discontinued therapy regained approximately two-thirds of the lost weight within six months - supporting the dose- and duration-dependent rebound patterns observed in our analysis. Similarly, Quarenghi et al. (2025) [14] conducted a randomized trial examining liraglutide withdrawal in patients with type 2 diabetes and reported a mean weight regain of 2.4 kg at 24 weeks post-discontinuation, corroborating the rebound trends identified in our subgroup analysis.

These patterns extend beyond GLP-1 receptor agonists. Khera et al. (2016) [54], through a network meta-analysis of various anti-obesity agents, found that weight regain was common across drug classes following treatment cessation, with the degree of rebound proportional to the initial weight loss achieved. Likewise, Greenway (2015) [7] reported rapid weight regain after cessation of lorcaserin, highlighting that the phenomenon is not drug-specific but likely reflects a broader pharmacologic class effect. Supporting this is the notion that long-term or indefinite pharmacotherapy may be necessary to counteract biological adaptations, such as hormonal and metabolic changes, that predispose individuals to weight recidivism - mechanisms consistent with the temporal weight regain trends observed in our review.

Furthermore, a position statement by Bray and Bouchard (2020) [55], on behalf of the Obesity Society, underscores that obesity is a chronic, relapsing condition requiring sustained therapy and ongoing weight monitoring. Together, these external sources reinforce the generalizability and clinical importance of our findings: (1) weight regain is a consistent outcome upon cessation of anti-obesity therapy, (2) the extent of rebound correlates with the initial magnitude of weight loss, and (3) long-term efficacy demands a multidisciplinary and continuous management strategy. Anti-obesity therapies should be viewed and discussed as long-term, potentially lifelong treatments - much like antihypertensive or lipid-lowering medications. Physicians should counsel patients to expect the possibility of weight regain after cessation and work collaboratively to develop maintenance plans that blend ongoing pharmacotherapy with evidence-based lifestyle, behavioral, and nutritional strategies. Furthermore, regular monitoring and personalized management - including readiness for dose adjustment, transition to alternative agents, or implementation of adjunctive interventions - are essential to mitigate relapse and sustain the health benefits of weight reduction.

Ultimately, these results reinforce the chronic, relapsing nature of obesity and call for a paradigm shift in both clinical practice and patient education. Obesity should be regarded and managed as a chronic, relapsing disease - akin to hypertension or diabetes - rather than a condition curable by short-term intervention. The observed weight regain following drug withdrawal reflects the underlying pathophysiology of energy homeostasis rather than therapeutic failure. Consequently, prolonged or stepped-down pharmacotherapy - combined with sustained lifestyle and behavioral interventions - represents the most effective strategy for durable weight control. By acknowledging and planning for the realities of weight regain, healthcare providers can better support patients in achieving meaningful, lasting improvements in weight and associated comorbidities.

This meta-analysis provides valuable insight into weight regain following the discontinuation of GLP-1 receptor agonists and other anti-obesity drugs; however, it is subject to several limitations that must be acknowledged. First, significant heterogeneity exists among included studies, particularly regarding treatment duration, drug type, dose, baseline population characteristics (such as age, BMI, or presence of diabetes), and follow-up periods post-discontinuation. This variability complicates direct comparisons and may affect the generalizability of pooled estimates. Second, most studies relied on intention-to-treat populations and may not fully account for attrition bias, as individuals who experience greater rebound may be more likely to drop out from follow-up, potentially underestimating true weight regain. Third, the majority of trials had relatively short post-discontinuation follow-up durations (often 3 to 12 months), limiting the understanding of long-term weight trajectories. Fourth, behavioral, dietary, and physical activity interventions were inconsistently reported, and may confound pharmacological effects, as sustained lifestyle support is known to influence weight maintenance outcomes. Fifth, few studies systematically assessed psychological or metabolic adaptations (such as changes in appetite hormones) that may underlie weight recidivism.

Future research should prioritize long-term, prospective randomized studies with standardized protocols for follow-up, detailed reporting on adjunct lifestyle interventions, and thorough assessment of patient subgroups to identify individuals most at risk for significant rebound. Mechanistic studies exploring biological drivers of weight regain, and real-world evidence from diverse populations, will also be critical in guiding optimal chronic obesity management and personalized therapy duration [12,56].

## Conclusions

While these drugs are undeniably effective in achieving initial weight loss, the data clearly indicate that their benefits are vulnerable to reversal once treatment is ceased. The magnitude of weight regain observed across multiple high-quality randomized controlled trials underscores the chronic and relapsing nature of obesity, reflecting underlying physiological compensatory mechanisms, such as hormonal dysregulation and reduced energy expenditure. Importantly, this analysis also highlights the drug-specific variability in rebound weight patterns, with GLP-1 receptor agonists, like semaglutide, showing greater initial efficacy but also more pronounced regain when discontinued. This paradox reinforces the necessity for clinicians to treat obesity not as a short-term condition but as a long-term, possibly lifelong disease requiring continuous pharmacologic and behavioral interventions. The findings support the urgent need for sustained maintenance strategies, such as prolonged or stepped-down pharmacotherapy, integration of nutritional and physical activity programs, and individualized patient follow-up to mitigate relapse. From a public health and policy perspective, these findings advocate for a paradigm shift in how obesity treatment is approached, funded, and monitored. Anti-obesity drugs should no longer be viewed as temporary solutions but rather as integral components of a chronic care model. Furthermore, future research should explore optimal tapering strategies, combination regimens, and biomarkers to predict individuals most at risk of rebound. By framing obesity as a lifelong disease, clinicians and policymakers can better support strategies that prioritize maintenance over temporary weight loss.

In conclusion, this study provides robust quantitative evidence that weight regain is not a treatment failure but a predictable biological response and must be anticipated and addressed in clinical practice. By reframing pharmacotherapy as a maintenance tool rather than a finite intervention, healthcare systems can better support patients in achieving sustained, long-term weight control and improved metabolic health.

## Figures and Tables

**Figure 8:**
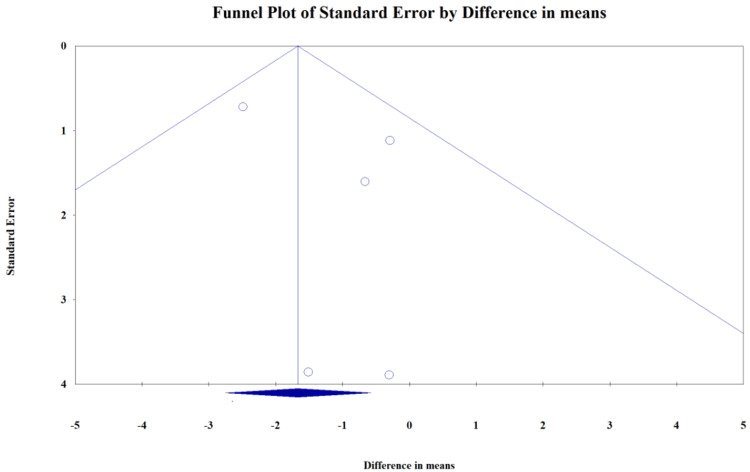
Funnel plots on the outcome orlistat weight regain

**Figure 9:**
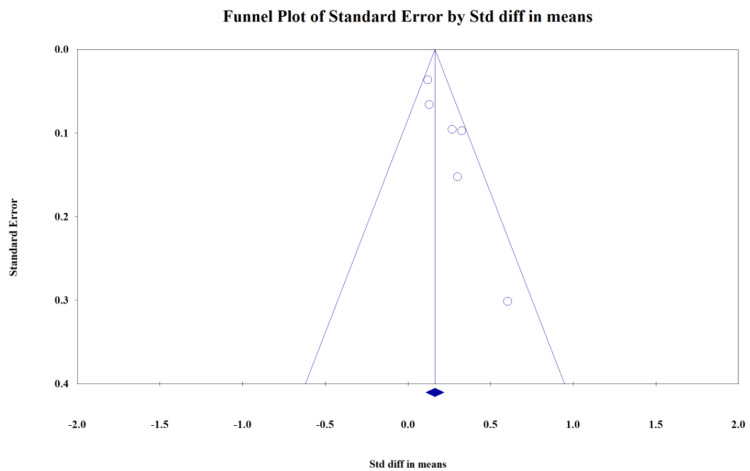
Funnel plots on the outcome liraglutide weight regain

**Figure 10:**
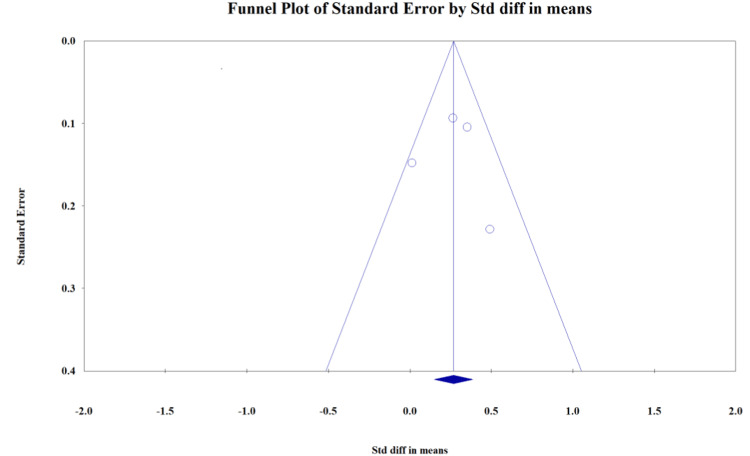
Funnel plots on the outcome exenatide weight regain

**Figure 11:**
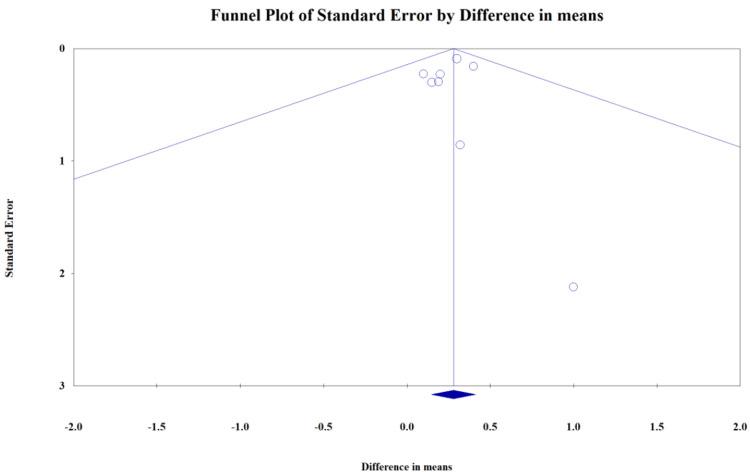
Funnel plots on the outcome semaglutide weight regain

**Figure 12:**
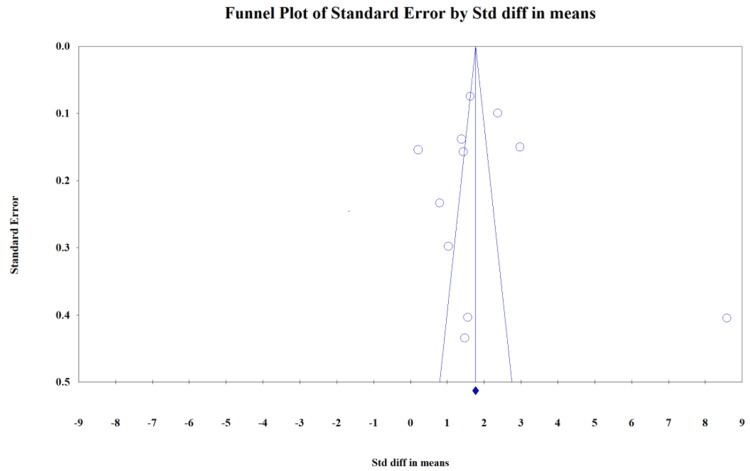
Funnel plots on the outcome weight regain post-discontinuation of anti-obesity medication

## Appendices

Funnel plots
